# Supplementary material for: The Effect of Alkyl Substitution of Novel Imines on Their Supramolecular Organization, towards Photovoltaic Applications
Source: Polymers (Basel). 2021 Mar 26;13(7):1043. doi: 10.3390/polym13071043 (PMC8036393; doi:10.3390/polym13071043)
Supplement: Supplementary file 1 [file polymers-13-01043-s001.pdf]

Supplementary Information

The effect of alkyl substitution of novel imines on their supramolecular organization, towards photovoltaic applications

Paweł Nitschke[1], Bożena Jarząbek[1*], Marharyta Vasylieva[1, 2], Marcin Godzierz[1], Henryk Janeczek[1], Marta Musioł[1], Adrian Domiński[1]

[1]Centre of Polymer and Carbon Materials, Polish Academy of Sciences, 34 M. Curie-Skłodowska Str., 41-819 Zabrze, Poland
bozena.jarzabek@cmpw-pan.edu.pl (B.J.); pnitschke@cmpw-pan.edu.pl (P.N.); mvasylieva@cmpw-pan.edu.pl (M.V.); mgodzierz@cmpw-pan.edu.pl (M.G.); hjaneczek@cmpw-pan.edu.pl (H.J.); mmusiol@cmpw-pan.edu.pl (M.M.); adominski@cmpw-pan.edu.pl (A.D.)
[2]Silesian University of Technology, Faculty of Chemistry, 9 Strzody Str., 44-100 Gliwice, Poland
*Correspondence: bozena.jarzabek@cmpw-pan.edu.pl;

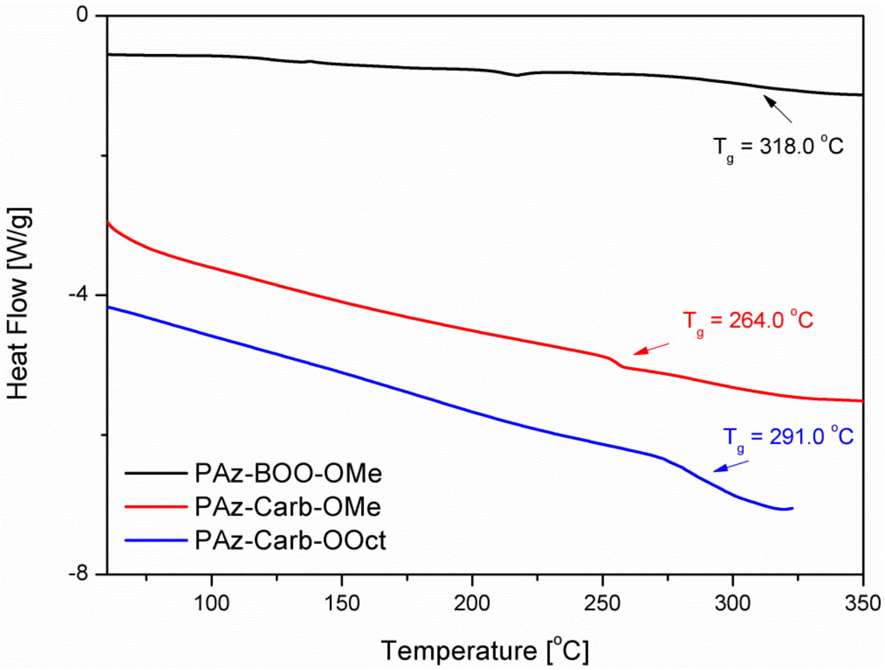

Figure S1. DSC curves obtained during the second heating stage of investigated compounds.

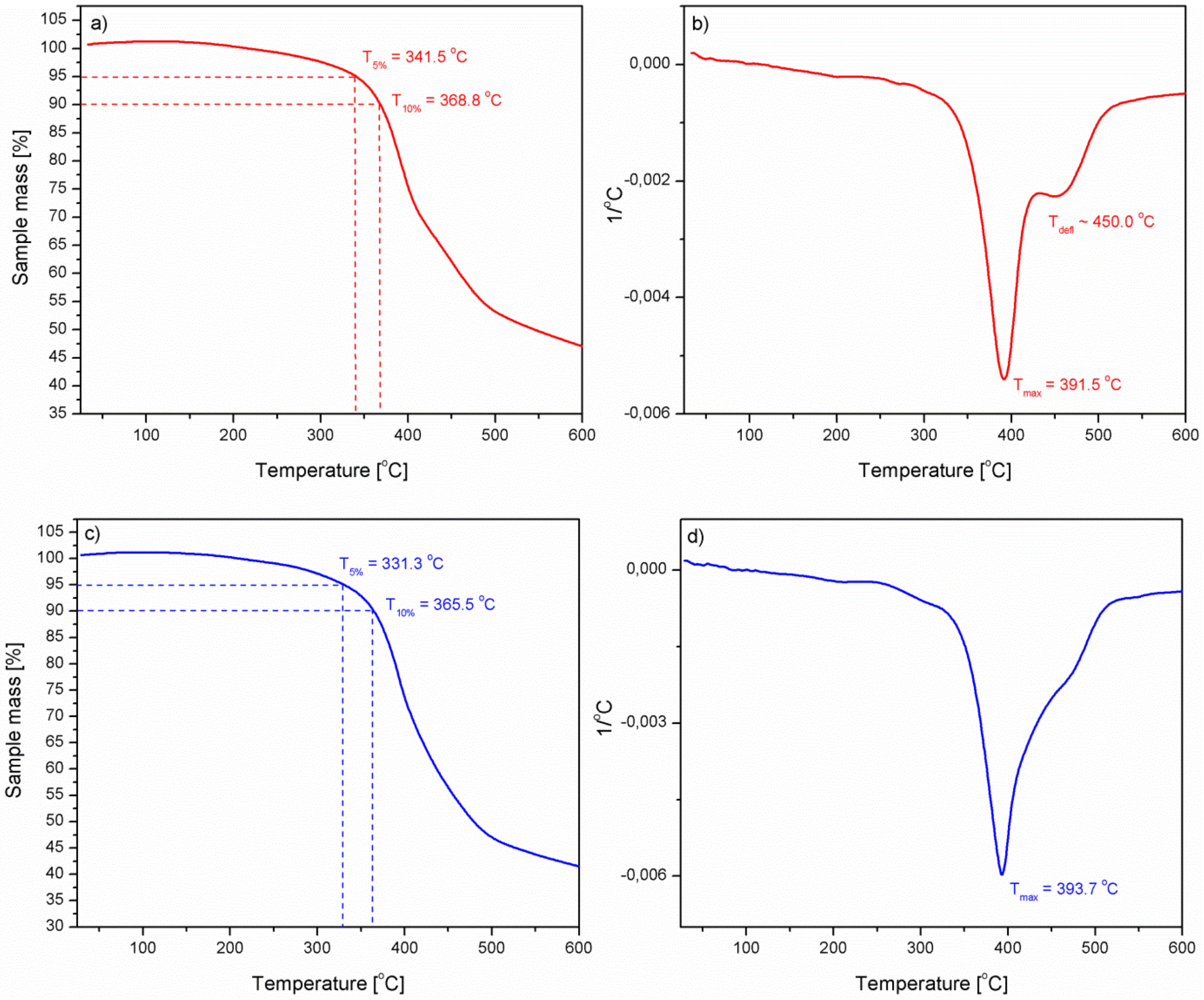

Figure S2. TGA (a, c) and DTG (b, d) curves of investigated compounds: PAz-Carb-OMe (red lines) and PAz-Carb-OOct (blue lines).
